# Prevalence of Maternal Postnatal Anxiety and Its Association With Demographic and Socioeconomic Factors: A Multicentre Study in Italy

**DOI:** 10.3389/fpsyt.2021.737666

**Published:** 2021-09-30

**Authors:** Loredana Cena, Antonella Gigantesco, Fiorino Mirabella, Gabriella Palumbo, Alice Trainini, Alberto Stefana

**Affiliations:** ^1^Department of Clinical and Experimental Sciences, Section of Neuroscience, Observatory of Perinatal Clinical Psychology, University of Brescia, Brescia, Italy; ^2^Center for Behavioural Sciences and Mental Health, National Institute of Health, Rome, Italy

**Keywords:** postnatal anxiety, obstetric factors, socioeconomic factors, demographic factors, psychological support

## Abstract

Anxiety is a common perinatal disorder that can cause severe adverse consequences. This study (a) assesses the prevalence of maternal postnatal anxious symptomatology, and (b) analyses its association with demographic and socioeconomic variables as well as obstetric and other psychosocial variables. The assessment included 307 mothers aged ≥18 years with a biological baby aged ≤ 52 weeks and from seven Italian healthcare centres, evaluated using a Psychosocial and Clinical Assessment Form (also covering demographic and socioeconomic factors), and the state scale of the State-Trait Anxiety Inventory. We found an overall prevalence of self-reported postnatal anxious symptoms of 34.2%. More specifically, the prevalence was 34.5% at 1–24 weeks postpartum, and 30.8% at >24 weeks postpartum. No associations between postnatal anxious symptoms and demographic or socioeconomic variables were observed. As regards the other variables, the findings indicated antenatal depression or anxiety, parity, and current psychological support from the partner as having the strongest relationships.

## Introduction

Anxiety is one of the most common postpartum mental disorders (1–3); anxiety is more common than depression, and these two conditions often occur concomitantly (1, 4). Postnatal anxiety may impact on mothers' own well-being and their children's health and development. For women themselves, these impacts include poorer quality of life and reduction in their abilities to perform daily activities and parenting, increased risk of chronic disease and substance abuse, as well as the economic burden of health care costs, loss of earnings, and unemployment (5). Moreover, postnatal anxiety can negatively affect breastfeeding (6) and early mother–infant interactions (7–11) at a time when children are most sensitive to their environments, resulting in poorer behavioral, cognitive, and emotional development outcomes for children (12–14). Nevertheless, anxiety has received less attention than it deserves in routine clinical practice and research, remaining largely undetected and untreated. Accordingly, the body of literature on the prevalence and risk factors of anxiety from the postpartum period is limited (14).

The few previous studies that investigated the association between demographic and socioeconomic factors and maternal postnatal anxiety (2, 14, 15) found that some demographic (e.g., age) and socioeconomic variables (e.g., education, employment, financial situation) were associated with anxious symptoms and/or anxiety disorders. However, the results are equivocal and the associations between anxiety and socioeconomic factors may change over time, especially during periods of major socio-political and economic change (16, 17), such as the current COVID-19 pandemic (18, 19). Other factors for which associations with anxiety are reported by more than one study were partner support (20–23), maternal self-efficacy (23, 24), and history of depression (20, 23, 25). Overall, despite the existing contributions to the field, the clear identification of specific risk factors affecting the occurrence of anxious symptomatology or anxiety disorders in women during the postnatal period remains an area of on-going research. Understanding the risk factors of postnatal anxiety can inform health policy to make more targeted and responsive health service system during the postpartum period and early months of raising children, providing mental health support for mothers both in the immediate postnatal period and throughout the early childhood months.

The aims of this study were: (a) to assess the prevalence of maternal postnatal anxious symptomatology and (b) to analyse the associations of postnatal anxious symptomatology with some demographic, socioeconomic, obstetric, interpersonal, and antenatal maternal mental health variables.

## Method

### Outline of the Study

This study was conducted as part of a multicentre, longitudinal study called the “Screening and early intervention for perinatal anxiety and depressive disorders: Prevention and promotion of mothers', children's, and fathers' mental health.” The study design was developed in mutual agreement of scientific collaboration between the University of Brescia, Department of Clinical and Experimental Sciences, Section of Neuroscience, Observatory of Perinatal Clinical Psychology (https://www.unibs.it/it/node/988) and the Italian National Institute of Health. Information about the rationale and methodology of the whole study were detailed in the study protocol (26). Ethical approval was granted by the Ethical Committee of the Healthcare Centre of Bologna Hospital (Register Number: 0077805, June 27, 2017). The Observatory of Perinatal Clinical Psychology (University of Brescia) managed the implementation of the study. All operational professionals involved in the study had completed postgraduate training in perinatal clinical psychology at the University of Brescia Observatory of Perinatal Clinical Psychology and had attended a course on screening, assessment, and treatment for maternal perinatal mental health problems, developed by the National Institute of Health (27).

### Study Protocol

This study only presents cross-sectional data because screening for anxious symptomatology was carried out at baseline.

Participants were recruited during a routine postnatal health check-up or pediatric vaccination appointment at one of seven publicly-funded healthcare facilities (located in (Bologna Child and Adolescent Neuropsychiatry, Brescia, Collegno [Turin], Florence, Mantua, Milan and Treviolo [Bergamo])) between 2017 and 2018. Consecutive women were invited to participate in the study. Specifically, the participation to the study was offered by the obstetricians or gynecologists or pediatricians of those facilities. All the women approached were provided with a pamphlet developed as part of the study, in which the purpose, aims, and methodology of the study were explained. Women who wished to participate provided their personal information (name or phone number to be contacted later) in order to meet up with trained clinical psychologists. Women who confirmed they wanted to be involved and definitively agreed to participate signed an informed consent form. Then, they were underwent a semi-structured interview led by trained clinical psychologists to elicit information on current and past maternal experience with psychiatric conditions and use of psychotropic drugs. The inclusion criteria to be enrolled were being able to speak and read Italian well and being a woman aged ≥18 years with a biological baby aged ≤ 52 weeks. The exclusion criteria were having issues with drug or substance misuse and/or having on-going psychotic symptoms. The enrolled women were then administered the pertaining scheduled self-report assessment instruments for data collection (see Assessment instruments). All the women completed the interviews and self-report instruments at the facilities; the majority of them on the same day in which they were invited to join. Few women provided their phone numbers to be contacted for arranging a subsequent appointment at the facilities in order to complete the instruments.

The sample size for this study was calculated using a single population proportion formula based on a 95% confidence level, an expected prevalence of 15% (1), and a precision of 0.05. The recommended sample size was 196 subjects.

### Assessment Instruments

All participants completed the postnatal version of the Psychosocial and Clinical Assessment Form (PCAF) (28) and the State-Trait Anxiety Inventory (STAI) (29).

The postnatal PCAF is composed of 23 items covering demographic and socioeconomic factors, maternal perceived partner support, maternal anxiety during pregnancy (having experienced a period of at least 6 consecutive months when woman felt apprehensive, anxious, easily worried about many things and more than usual during her last pregnancy), maternal depressed mood during pregnancy (having experienced a period of at least 2 consecutive weeks in which nearly every day and for most of the day woman felt sad, blue, or depressed during her last pregnancy) and other obstetric information regarding pregnancy, pre-delivery, and delivery as shown in Table 1.

**Table 1:** Characteristics of the sample and prevalence of current postnatal anxiety.

	**Sample** ***N* (%)**	**STAI ≥40** ***N* (%)**
**Age**		
18–29	50 (16.3)	18 (36.0)
30–35	133 (43.5)	40 (30.1)
>35	123 (40.2)	47 (38.2)
Nationality^***^		
Italian	283 (92.2)	93 (32.9)
Non-Italian	24 (7.8)	12 (50.0)
**Marital status**		
Married or cohabiting	275 (90.5)	91 (33.1)
Single, separated, divorced, or widowed	29 (9.5)	13 (44.8)
Educational level^**^		
University	138 (45.5)	57 (41.3)^*^
Secondary	118 (38.9)	31 (26.3)
Primary or Illiterate	47 (15.5)	16 (34.0)
**Working status**		
Permanent employee	210 (69.3)	70 (33.3)
Temporary employee	24 (7.9)	9 (37.5)
Student, homework, or unemployed	69 (22.8)	26 (37.7)
**Economic status**		
Average high status	122 (40.4)	37 (30.3)
A few problems	152 (50.3)	54 (35.5)
Some or many problems	28 (9.3)	13 (46.4)
Planned pregnancy^***^		
Yes	233 (76.4)	74 (31.8)
No	72 (23.6)	31 (43.1)
**Resort to assisted reproductive technology**		
Yes	25 (8.2)	9 (36.0)
No	280 (91.8)	96 (34.3)
Parity^***^		
Multiparous (>1 child)	130 (42.3)	38 (29.2)
Primiparous	177 (57.7)	67 (37.9)
**Past abortion**		
Yes	89 (29.5)	31 (34.8)
No	213 (70.5)	73 (34.3)
Complication during childbirth^***^ (e.g., elective cesarean, emergency cesarean, excess blood loss, vaginal tearing, adverse effects of epidural analgesia, forceps, >36-h labor, preterm birth and low birth weight, emotional distress during labor, or delivery)		
Yes	84 (27.7)	35 (41.7)
No	219 (72.3)	70 (32.0)
**Children living in the time of this last pregnancy**		
Yes	113 (36.8)	32 (28.3)
No	194 (63.2)	73 (37.6)
**Breastfeeding**		
Yes	243 (81.8)	89 (36.6)
No	54 (18.2)	15 (27.8)
Anxiety during pregnancy^*^		
Yes	49 (16.4)	35 (71.4)
No	250 (83.6)	67 (26.8)
Depressed mood during pregnancy^*^		
Yes	68 (22.4)	49 (72.1)
No	235 (77.6)	56 (23.8)
Counting on husband/partner when woman feels nervous or worried^*^		
Not at all or a little	54 (17.8)	33 (61.1)
Enough	82 (27.0)	31 (37.8)
A lot	168 (55.3)	40 (23.8)
Total	307 (100.0)	105 (34.2)

* *p ≤ 0.001,*

** *p ≤ 0.05, and*

*** *p ≤ 0.10 difference between women with and without postnatal anxiety resulted in the univariate analysis (Chi2 test)*.

The STAI is a self-rating scale containing 40 items divided into two subscales evaluating state anxiety (i.e., anxiety in the current situation or time period) and trait anxiety (i.e., relatively stable subjective aspects of propensity toward elevated levels of anxiety). The total score for each subscale ranges from 20 to 80, with higher scores indicating more severe anxious symptomatology. The STAI internal consistency range is 0.86–0.95 (29, 30) and the Italian validation (on a non-pregnant population) (31) showed psychometric properties consistent with those measured in the original version. This study only considered the state anxiety subscale and a cut-off score of ≥40 was adopted as recommended for the postpartum period (32).

### Statistical Analysis

Two prevalence estimates of anxiety were calculated, respectively, from women assessed during the first 24 weeks postpartum and women assessed after. The characteristics or risk factors of women with current anxiety and women without were summarized using descriptive statistics. The Chi2 test was used to test for differences between the two groups of women. After univariate estimations were calculated, a multiple logistic regression model was constructed in which current anxiety served as the dependent variable, while a number of risk factors measures with *p* < 0.10 in the univariate analyses were entered as independent variables. All analyses were performed using the Statistical Package for Social Science (SPSS) version 26 (SPSS Inc., Chicago, IL).

## Results

A total of 307 new mothers participated in the study. Twenty-nine per cent of women invited to join the study refused to participate. The characteristics of the participant women are reported in Table 1.

The overall prevalence of self-reported postnatal non-specific anxious symptoms was 34.2%. More specifically, the prevalence was 34.5% (*n* = 97 of 281) at 1–24 weeks postpartum, and 30.8% (*n* = 8 of 26) at >24 weeks postpartum.

Univariate analyses (Table 1) showed a significantly higher risk of anxiety in mothers who had depression or anxiety during pregnancy (*p* < 0.001), lack or enough psychological support from the partner (*p* < 0.001), and high educational level (university degree or above) (*p* < 0.05). No statistically significant associations were observed between anxiety and economic variables (i.e., economic status and working status).

The logistic regression analysis (Table 2) finally indicated that having suffered from anxiety or depression during pregnancy was positively associated with postnatal anxiety, whereas being multiparous and being able to count a lot on own partner for emotional support were inversely associated with postnatal anxiety.

**Table 2:** Risk factors associated with current symptoms of postnatal anxiety as assessed with STAI (≥40): results of multiple logistic regression analysis.

**Variable**	**Categories**	**Adjusted odds ratio (95% CI)**	***P*** **-value (Wald-test)**
Nationality	Italian	1.00	
	Non-Italian	1.60 (0.53–4.81)	0.405
Educational level	Primary or illiterate	1.00	
	Secondary	0.89 (0.35–2.28)	0.811
	University	1.98 (0.81–4.82)	0.134
Planned pregnancy	No	1.00	
	Yes	0.69 (0.35–1.37)	0.292
Complication during childbirth	No	1.00	
	Yes	1.60 (0.85–3.04)	0.148
Parity	Primiparous	1.00	
	Multiparous (>1 child)	0.33 (0.17–0.63)	0.001
Anxiety during pregnancy	No	1.00	
	Yes	3.48 (1.52–7.98)	0.003
Depressed mood during pregnancy	No	1.00	
	Yes	4.67 (2.26–9.66)	0.000
Counting on husband/partner when woman feels nervous or worried	Not at all or a little	1.00	
	Enough	0.44 (0.18–1.06)	0.067
	A lot	0.26 (0.11–0.63)	0.003

## Discussion

The prevalence of postnatal anxiety found in this study was more than double the overall pooled prevalence of 15.0% (at 1–24 weeks postpartum) and 14.8% (>24 weeks) reported by meta-analytic studies (1). Differences in the prevalence of maternal postnatal anxiety (which also remained when the same diagnostic tool was used) could be due to (a) particular individual and clinical characteristics of participants and (b) the fact that one of the major reasons for individuals' refusal to participate in the current study was women feeling that they were in good health and confident that they would not become anxious or depressed. With regard to the use of the STAI for this study, it should be kept in mind that the validity and reliability aspects of this instrument [i.e., discriminant and predictive validity, short-term test-retest reliability; (33)] and its easy and quick use can yield a reasonably accurate estimate of prevalence, while its widespread utilization in research (1, 34) allows for accurate comparisons among countries.

Twenty-four weeks was chosen for descriptive purposes as reference period, in order to make it possible comparing our prevalence findings with those of Dennis et al. (1) who used that period in their meta-analysis on the prevalence of postnatal maternal anxiety. Regarding the 1–24 weeks prevalence of postnatal anxiety, we found that the prevalence of maternal anxious symptoms was high at 1–24 weeks postpartum, and then decreased slightly after 24 weeks postpartum. This observation is consistent with the results previously found, indicating a lower and steadier rate of decline in maternal anxious symptoms in the postpartum period (1). However, it should be noted that the findings on the monthly/trimestral/biannual prevalence of perinatal anxiety are not univocal (1, 2).

The present study showed that antenatal history of depression or anxiety increased the risk of postnatal anxiety symptoms, confirming previous findings (20, 23, 25). Further, the protective effect against postnatal anxiety of perceiving a lot of psychological support from the partner was in accordance with previous comparable studies which reported that perceived lack of social support from the partner is an important risk factor for postnatal anxiety (21, 23, 24). We also found that being multiparous was associated with a lower risk of postnatal anxiety, not confirming some previous findings which showed that there was association between anxiety symptoms and multiparity, although only among multiparous women who had simultaneously a psychiatric history or high levels of stress (24). Our opinion is that our finding suggests that, in general, multiparous women are more skilled in the exercise of parenting and this may enhance their self-efficacy which in turn is protective against anxiety (23). Complication during birth was not found to be a risk factor in the present study; this is consistent with the few available comparable studies (20, 23, 24). Other variables such as nationality and unplanned pregnancy, which were not found to be risk factors, could not be compared as, to our knowledge, they were not included in comparable studies.

Interestingly, using a sample of pregnant women from the same larger study, we found that there was a significantly higher risk of anxiety in pregnant women with poor education, who were unemployed and who had financial problems (35). Additionally, pregnant women experienced higher level of anxious symptoms when the pregnancy was unplanned, they had a history of abortion or had other children at the time of the pregnancy (35).

However, and this is the interesting thing, all the risk factors for maternal anxiety that are identified during pregnancy do not apply during the postpartum period. This is inconsistent with a number of previous studies that showed that poor education and worst economic condition were common risk factors for maternal antenatal anxiety (2, 14, 15), although some other studies reported that, at least as regards to poor education, the risk was not increased (20, 24). One could argued that in the present study the lack of relationships between poor education and worst economic condition and current anxiety are likely to be underestimated because the sample variability is restricted, given that the sample was mostly made up of women with no financial difficulties and high education. It should be noted, however, that education and economic conditions of women in the present study are comparable to those of pregnant women from the larger study, although the women in the present study partially differ from those of the pregnant study with regards to age (in the present study, 40% were aged 35 years or more, vs. 29% in the pregnant sample), previous pregnancies (42 vs. 25%) and presence of children living at the time of pregnancy (37 vs. 17%). This might suggest that economic and education conditions are more likely to become risk factors among younger and inexpert women (i.e., with a lower number of previous pregnancies or children living at the time of the last pregnancy). However, this suggestion should be interpreted with caution because we cannot exclude the possibility of lack of significant relationships due to the scarce variability and the not very large size of the sample (as compared with the sample of 1,142 pregnant women) (36), which may fail to detect significant differences that are really present.

In any case, the short- and long-term adverse financial and employment effects of the on-going COVID-19 pandemic and measures adopted to prevent and/or contain its spread (37, 38) make necessary new research projects aimed to investigate the role played by the socioeconomic condition of perinatal women and their families in the onset, duration, remission, and recurrence of perinatal anxiety. Indeed, the COVID-19 pandemic has negatively and significantly impacted national and global economies both in the present and in the coming years (18, 19). Furthermore, as shown by general as well as perinatal population surveys, social isolation related to the COVID-19 pandemic is associated with a wide range of adverse psychological effects, including clinical anxiety and concerns about lack of employment and financial difficulties (39–41), which could persist for years after the crisis (42). New mothers represent a vulnerable population and therefore may be among those most affected.

Four main limitations of this study should be mentioned. First, a cross-sectional approach to assessing perinatal anxiety does not enable us to fully and accurately explore whether (and how) the anxiety begins during pregnancy and progresses to the postnatal period. Second, the level of maternal anxious symptoms was based on self-reported measurements. Third, only 8.5% of our sample was composed by women at >24 weeks postpartum, this low percentage is not representative of all women between 26th and 52th weeks postpartum. Fourth, the sample had scarce variability with regards to educational level and economic condition.

Nevertheless, this article reveals important findings. In particular, high anxious symptoms are common among new mothers, significantly more common than depressive symptoms (36, 43, 44). The results of this study will serve as a baseline for future comparisons between Western countries, as well as for the abovementioned future research on the prevalence/incidence of postnatal anxiety and its protective and risk factors, especially during the current COVID-19 pandemic period.

## Data Availability Statement

The complete dataset is available from the corresponding author upon reasonable request.

## Ethics Statement

The studies involving human participants were reviewed and approved by Ethical Committee of the Healthcare Centre of Bologna Hospital (Register Number: 0077805, June 27, 2017). The patients/participants provided their written informed consent to participate in this study.

## Author Contributions

LC and GP contributed equally to the general study design. LC and AT from the Observatory of Perinatal Clinical Psychology coordinate and manage the implementation of the study in each healthcare centers. FM and AS designed the plan of statistical analysis of the study. AG serves primarily as research statistical analysis supervisor. AS, LC, and FM participated in the writing of the manuscript. GP revised the manuscript. AG designed a new plan of statistical analysis according to the requests of the reviewers and revised the first version of the manuscript. FM participated in realizing the new statistical analyses. All authors have critically reviewed and agreed this final version of the article.

## Funding

This work was funded by the Observatory of Perinatal Clinical Psychology, Department of Clinical and Experimental Sciences, Section of Neuroscience, University of Brescia (Italy).

## Conflict of Interest

The authors declare that the research was conducted in the absence of any commercial or financial relationships that could be construed as a potential conflict of interest.

## Publisher's Note

All claims expressed in this article are solely those of the authors and do not necessarily represent those of their affiliated organizations, or those of the publisher, the editors and the reviewers. Any product that may be evaluated in this article, or claim that may be made by its manufacturer, is not guaranteed or endorsed by the publisher.
